# Cohort study to evaluate prognostic factors in idiopathic pulmonary fibrosis patients introduced to oxygen therapy

**DOI:** 10.1038/s41598-023-40508-8

**Published:** 2023-08-22

**Authors:** Kensuke Kataoka, Keishi Oda, Hajime Takizawa, Takashi Ogura, Atsushi Miyamoto, Yoshikazu Inoue, Shinobu Akagawa, Seishu Hashimoto, Tomoo Kishaba, Koji Sakamoto, Naoki Hamada, Kazuyoshi Kuwano, Masayuki Nakayama, Masahito Ebina, Noriyuki Enomoto, Yasunari Miyazaki, Kenichiro Atsumi, Shinyu Izumi, Yoshinori Tanino, Hiroshi Ishii, Hiroshi Ohnishi, Takafumi Suda, Yasuhiro Kondoh

**Affiliations:** 1 Department of Respiratory Medicine and Allergy, Tosei General Hospital, 160 Nishioiwake-cho, Seto, Aichi 489-8642 Japan; 2 Department of Respiratory Medicine, University of Occupational and Environmental Health, Kitakyushu, Fukuoka Japan; 3 Department of Respiratory Medicine, Kyorin University School of Medicine, Mitaka, Tokyo Japan; 4 Department of Respiratory Medicine, Kanagawa Cardiovascular and Respiratory Center, Yokohama, Kanagawa Japan; 5 Department of Respiratory Medicine, Respiratory Center, Toranomon Hospital, Tokyo, Japan; 6 Clinical Research Center, National Hospital Organization Kinki-Chuo Chest Medical Center, Sakai, Osaka Japan; 7 Department of Respiratory Medicine, National Hospital Organization Tokyo Hospital, Tokyo, Japan; 8 Department of Respiratory Medicine, Tenri Hospital, Tenri, Nara Japan; 9 Department of Respiratory Medicine, Okinawa Chubu Hospital, Uruma, Okinawa Japan; 10 Department of Respiratory Medicine, Nagoya University Graduate School of Medicine, Nagoya, Aichi Japan; 11 Graduate School of Medical Sciences, Kyushu University, Research Institute for Diseases of the Chest, Fukuoka, Japan; 12 Division of Respiratory Diseases, Department of Internal Medicine, The Jikei University School of Medicine, Tokyo, Japan; 13 Department of Internal Medicine, Division of Pulmonary Medicine, Jichi Medical University, Shimono, Tochigi Japan; 14 Department of Respiratory Medicine, Tohoku Medical and Pharmaceutical University Medical School, Sendai, Miyagi Japan; 15 Department of Internal Medicine, Second Division, Hamamatsu University School of Medicine, Hamamatsu, Shizuoka Japan; 16 Department of Respiratory Medicine, Tokyo Medical and Dental University, Tokyo, Japan; 17 Department of Pulmonary Medicine and Oncology, Graduate School of Medicine, Nippon Medical School, Tokyo, Japan; 18 Department of Respiratory Medicine, National Center for Global Health and Medicine, Tokyo, Japan; 19 Department of Pulmonary Medicine, Fukushima Medical University School of Medicine, Fukushima, Japan; 20 Department of Respiratory Medicine, Fukuoka University Hospital, Fukuoka, Japan; 21 Department of Respiratory Medicine and Allergology, Kochi Medical School, Kochi University, Nankoku, Kochi Japan

**Keywords:** Prognostic markers, Respiratory tract diseases

## Abstract

While high-level evidence is lacking, numerous retrospective studies have depicted the value of supplemental oxygen in idiopathic pulmonary fibrosis (IPF) and other interstitial lung diseases, and its use should be encouraged where necessary. The clinical course and survival of patients with IPF who have been introduced to oxygen therapy is still not fully understood. The objective of this study was to clarify overall survival, factors associated with prognosis, and causes of death in IPF patients after the start of oxygen therapy. This is a prospective cohort multicenter study, enrolling patients with IPF who started oxygen therapy at 19 hospitals with expertise in interstitial lung disease. Baseline clinical data at the start of oxygen therapy and 3-year follow-up data including death and cause of death were assessed. Factors associated with prognosis were analyzed using univariable and multivariable analyses. One hundred forty-seven eligible patients, of whom 86 (59%) were prescribed ambulatory oxygen therapy and 61 (41%) were prescribed long-term oxygen therapy, were recruited. Of them, 111 died (76%) during a median follow-up of 479 days. The median survival from the start of oxygen therapy was 537 ± 74 days. In the univariable analysis, low body mass index (BMI), low forced vital capacity (FVC), low diffusion capacity (D_LCO_), resting hypoxemia, short 6 min-walk distance, and high COPD assessment test (CAT) score were significantly associated with poor prognosis. Multivariable analysis revealed low BMI, low FVC, low D_LCO_, low minimum SpO_2_ on 6MWT, and high CAT score were independent factors for poor prognosis. The overall survival of IPF patients after starting oxygen therapy is about 1.5 years. In addition to pulmonary function tests, 6MWT and patient reported outcomes can be used to predict prognosis more accurately.

Clinical Trial Registration: UMIN000009322.

## Introduction

IPF is a specific form of chronic, progressive fibrosing interstitial pneumonia of unknown cause occurring in adults. It is characterized by a progressive decline of pulmonary function, worsening dyspnea, impaired health-related quality of life, and poor prognosis with a median untreated survival of 3–5 years from diagnosis^1,2^. One of the most important non-pharmacologic therapies in IPF is supplemental oxygen therapy. Current guidelines recommend that patients with IPF and clinically significant resting hypoxemia should be treated with long-term oxygen therapy (LTOT), which is based on very low-quality evidence^1,3–6^. For adults with interstitial pneumonia who have severe exertional room air hypoxemia without resting hypoxemia, ATS guideline suggests prescribing ambulatory oxygen therapy (AOT), with low-quality evidence^7^. Frequently, AOT is prescribed for the management of episodic breathlessness and with the expectation of improved exercise capacity^3,8–12^. There is no guidance on the use of AOT for patients with IPF and data on the proper timing, purpose, type, and prognosis of oxygen therapy in patients with IPF are limited.

In recent years, the importance of discussing end-of-life care for IPF patients has been emphasized. Indeed, the initiation of oxygen therapy is recognized as an appropriate trigger for advance care planning^13^. In conducting precise advance care planning (ACP) it is important to identify overall survival and prognostic factors for IPF patients after the initiation of oxygen therapy. We think that the prognostic factors at that time may differ depending on the degree of progression of the disease. Therefore, we conducted this cohort study to clarify overall survival, cause of death, and factors associated with the prognosis of IPF patients after starting oxygen therapy.

## Material and methods

### Study population and design

This registry was designed to survey data from patients with IPF who were started on supplemental oxygenation therapy at home (UMIN000009322). Participants were recruited from 19 Japanese hospitals with expertise in interstitial lung disease between December 2012 and November 2015. The inclusion criteria were as follows: a diagnosis of IPF, age > 18 years, and home oxygen therapy being started for the first time. We included not only LTOT but also AOT used only during exertion. We did not include nocturnal only oxygen therapy without daytime use. The diagnoses of IPF were made by the participating investigators based on the 2011 IPF international guidelines^1^. In Japan, the indications for oxygen therapy for patients with chronic respiratory failure are based on the following criteria: 1) arterial partial pressure of oxygen (PaO_2_) ≦ 55mmHg (7.3 kPa) or oxygen saturation as measured by pulse oximetry (SpO_2_) ≦ 88%; 2) PaO_2_ = 56–59 mmHg (7.5–7.9 kPa) or SpO_2_ = 89% plus signs of cor pulmonale or pulmonary hypertension. This study included not only patients who necessarily met these inclusion criteria, but also those who did not meet the criteria were included at the discretion of physicians at specialized facilities. The decision to start home oxygen therapy and the type of oxygen therapy depended on each site’s physicians. To exclude reasons for oxygen induction other than IPF, patients whose condition was unstable, or who had a comorbidity likely to affect survival at the start of supplemental oxygenation therapy, such as severe heart failure, recent pulmonary embolism, or advanced malignancy, were excluded.

In this study, LTOT was defined as a prescription of oxygen for ≥ 15 h per day, and AOT was defined as a prescription < 15 h per day.

The clinical data were collected at the time oxygen therapy was started (baseline). The subject baseline characteristics included age, sex, body mass index (BMI), smoking history, experience of surgical lung biopsy, duration of IPF, past history of acute exacerbation, forced vital capacity (FVC), diffusing capacity of the lung for carbon monoxide (D_LCO_), presence of pulmonary hypertension, resting hypoxemia (oxygen saturation of peripheral blood (SpO_2_) < 88%), arterial partial pressure of carbon dioxide (P_a_CO_2_), 6-min walk test (6MWT) distance, minimum SpO_2_, modified Medical Research Council Dyspnea (mMRC) scale, and Gender Age and Physiology (GAP) stage^14^. The diagnosis of pulmonary hypertension in this study was defined as mean pulmonary artery pressure ≥ 25 mmHg measured by right heart catheter, or when the following echocardiography criteria were met. On echocardiography, a diagnosis of pulmonary hypertension was made if the tricuspid regurgitation peak velocity (TRV) was 2.9–3.4 m/s with other signs of pulmonary hypertension or if the TRV was > 3.4 m/s^15^. Patient reported outcomes were measured by COPD Assessment Test (CAT), which has been successfully used in patients with interstitial lung disease in past studies^16,17^. Follow-up data (date of death and cause of death) were collected 3 years after the last case enrollment. The cause of death was determined by the investigators at each site after receiving the medical records and death certificate.

### Statistical analysis

Data for continuous variables are presented as median (interquartile range) or n (%). Overall survival was analyzed from the time oxygen therapy was started to either death by any cause or lung transplantation. The survival rate was estimated by the Kaplan–Meier method. Univariable Cox proportional regression was used to find prognostic factor candidates. Variables that showed a significant result univariately (*p* < 0.1) were included in the corresponding multivariable analysis. Given the correlation between mMRC and CAT established in previous studies on COPD^18^, we prospectively decided not to include mMRC as a variable in the multivariable analysis to avoid potential issues of multicollinearity. In this study, the composite variable, GAP index, was not employed in the multivariate analysis because gender, age, FVC, and DLCO were evaluated as variables. Additionally, as D_LCO_ was often not measurable, we created another model that did not include it as a factor in the multivariate analysis. We planned to accumulate data on 120 deaths to evaluate up to 12 prognostic factors. According to a previous report^19^, the 3-year mortality rate of IPF patients who were started on oxygen therapy was 80%, and the sample size was calculated to be 150 patients. Independent prognostic factors were identified in the multivariable Cox proportional hazard analysis. To compare AOT and LTOT, survival was assessed using a Cox proportional hazard model with extracted independent prognostic factors as covariates. In all study analyses, *p* < 0.05 was considered significant. All statistical calculations were performed using SPSS version 25.0 (SPSS Inc., Chicago, IL, USA).

### Ethics approval

This study was performed in accordance with the Declaration of Helsinki. The study was approved by the human-research review board at Tosei General Hospital (Tosei 301) and each site, and all patients provided written informed consent.

## Results

A total 147 IPF patients were included in the study, and the median length of follow-up was 479 days for all patients. Characteristics of the study population at the baseline are summarized in Table 1. The majority of patients were male (80%) with a median duration of IPF before enrollment of 46 months. Twenty-two (15%) patients had hypoxemia (SpO_2_ < 88%) at rest, and 124 (84%) had hypoxemia (SpO_2_ < 90%) on exertion. Of the 53 patients diagnosed with pulmonary hypertension, 9 were diagnosed by right heart catheter and the remaining 44 were diagnosed by echocardiography alone. Eighty-six (59%) patients were prescribed AOT and 61 (41%) were prescribed LTOT (Supplementary Table 1).

**Table 1:** Patient characteristics at initiation of oxygenation therapy.

Characteristic	Median (IQR) or n (%)
Age, years	72 (66–77)
Male	117 (80)
Body mass index, kg/m^2^	22.2 (19.8–25.0)
Smoking history	113 (77)
Surgical lung biopsy	32 (22)
Duration of IPF, months	46 (24–72)
Past history of acute exacerbation	28 (19)
FVC, % predicted	61.2 (51.3–72.2)
^#^D_LCO_, % predicted	39.6 (32.0–51.7)
Pulmonary hypertension	53 (36)
Resting hypoxaemia (SpO_2_ < 88%)	22 (15)
Resting PaCO_2_ on air, mmHg	40.7 (37.6–45.2)
6MWT distance, m	287 (190–401)
Minimum SpO_2_ on 6MWT, %	82 (72–87)
Meet either of the adaptation criteria, %	88 (60)
CAT	21 (15–27)
mMRC scale, 0/1/2/3/4	3 (2) / 18 (12) / 53 (36) / 56 (38) / 17 (12)
GAP stage, I/II/III	23 (16) / 119 (81) / 5 (3)
Comorbidities	
Cardiovascular disease	76 (52)
Cerebrovascular disease	6 (4)
Diabetes	38 (26)
Malignant disease	12 (8)
Psychiatric disease	4 (3)
Others	23 (16)

Data are presented as median (IQR) or n (%).

IQR, interquartile range; IPF, idiopathic pulmonary fibrosis; FVC, forced vital capacity; D_LCO_, diffusion capacity of the lung for carbon monoxide; SpO_2_, oxygen saturation of peripheral blood; P_a_CO_2_, arterial partial pressure of carbon dioxide; 6MWT, six-minute walk test; CAT, COPD assessment test; mMRC scale, modified Medical Research Council Dyspnea scale.

^#^n = 115.

Eighty-eight (60%) patients met at least one criterion for the introduction of oxygen therapy, while the remaining 59 (40%) did not meet any of the criteria. Patients who did not meet any of the criteria had hypoxemia on exertion and a higher CAT score, as did patients who met the criteria. There was no difference in prognosis between those who met the criterion and those who did not (Table 2).

**Table 2:** Comparing whether the criteria for introducing oxygen therapy are met or not.

	Meet the criteria for introducing oxygen therapy			
	Yes	No	*p*-value	
Patients	88 (59.9)	59 (40.1)		
Patient characteristics at initiation of oxygenation therapy				
Age, year	72.5 (67.0–78.8)	71.0 (66.0–76.0)	0.338	
Sex, male	72 (81.8)	45 (76.3)	0.414	
Body mass index, kg/m^2^	22.2 (19.8–24.9)	22.2 (19.6–25.0)	0.828	
Smoking history	65 (73.9)	47 (79.7)	0.438	
Duration of IPF, month	46.5 (24.0–74.3)	45 (26–66)	0.539	
Past history of acute exacerbation	19 (21.6)	9 (15.3)	0.396	
Surgical lung biopsy	19 (21.6)	45 (76.3)	0.841	
FVC, % predicted	60.7 (49.9–72.3)	62.0 (55.3–72.3)	0.513	
D_LCO_, %predicted	38.9 (32.0–48.0)	40.0 (29.5–52.2)	0.902	
Pulmonary hypertension	53 (60.2)	0 (0)	< 0.001	
Resting hypoxaemia (SpO_2_ < 88%)	22 (25.0)	0 (0)	< 0.001	
PaCO_2_ breathing air, mmHg	40.6 (37.6–45.5)	41.0 (37.7–45.0)	0.652	
6MWT distance, m	270 (171–385)	298 (200–452)	0.215	
Minimum SpO_2_ on 6MWT, %	80.0 (70.0–86.0)	83.5 (73.0–88.0)	0.068	
CAT	20.0 (15.0–26.0)	21.0 (16.0–28.0)	0.264	
mMRC scale				0.283
0	1 (1.1)	2 (3.4)		
1	9 (10.2)	9 (15.3)		
2	31 (53.2)	22 (37.3)		
3	39 (44.3)	17 (28.8)		
4	8 (9.1)	9 (15.3)		
GAP stage				0.141
I	12 (13.6)	11 (18.6)		
II	71 (80.7)	48 (81.4)		
III	5 (5.7)	0 (0)		
Outcome				
Median survival time, days	450 (259–641)	662 (420–903)	0.116	
Death	67	46	0.636	
Chronic respiratory failure	34 (50.7)	25 (54.3)		
Acute exacerbation	19 (28.4)	12 (26.1)		
Lung cancer	3 (4.4)	2 (4.3)		
Others	11 (16.4)	7 (15.2)		
Lung transplantation	1	0	N/A	

Data are presented as median (IQR) or n (%).

IQR, interquartile range; FVC, forced vital capacity; D_LCO_, diffusion capacity of the lung for carbon monoxide; SpO_2_, oxygen saturation of peripheral blood; PaCO_2_, arterial partial pressure of carbon dioxide; 6MWT, six-minute walk test; CAT, COPD assessment test; mMRC scale, modified Medical Research Council Dyspnea scale; GAP, gender age and physiology; N/A, not applicable.

**Figure 1:**
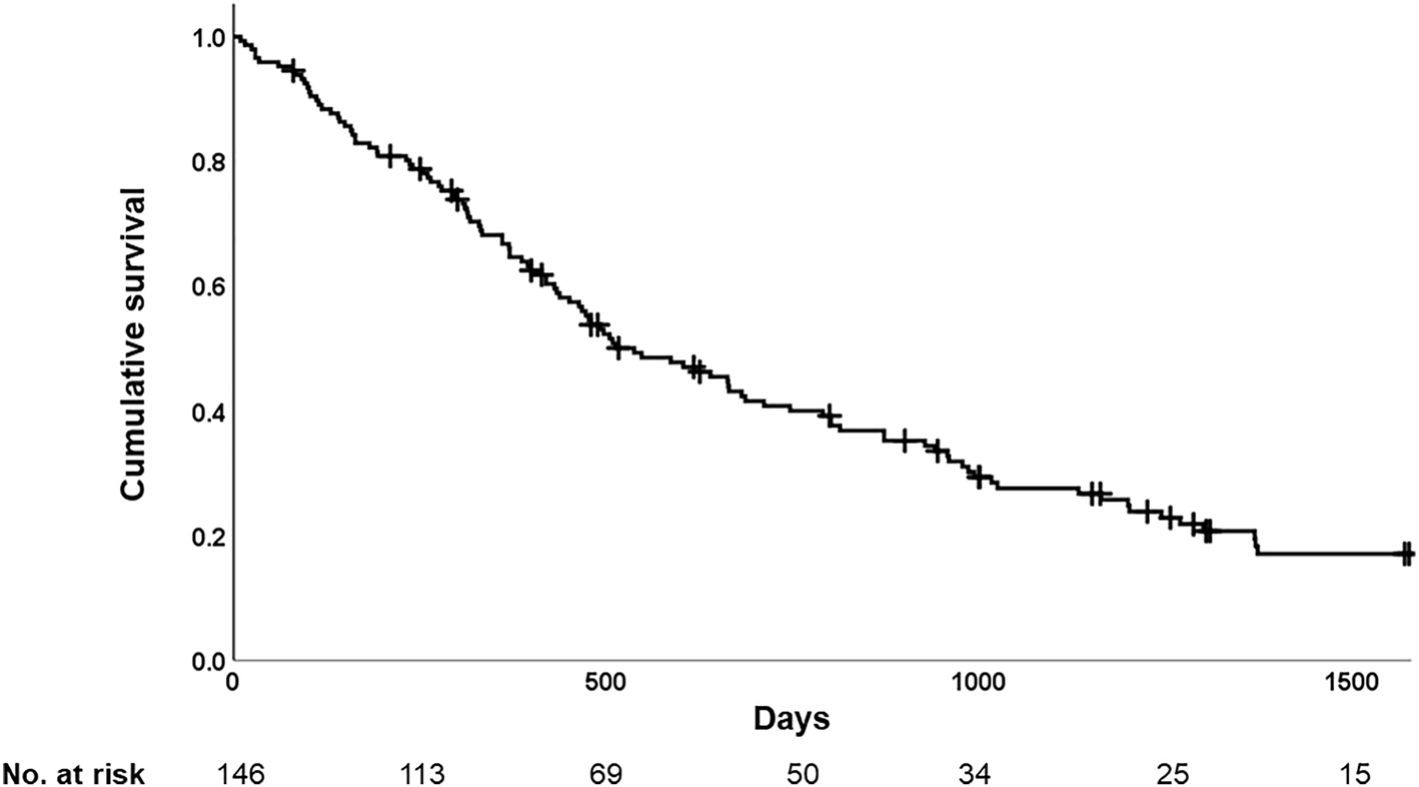
Survival time from the start of oxygen therapy. Median survival time was 537 ± 74 days.

During the observation period, 113 patients died, and one underwent lung transplantation. Causes of death were chronic respiratory failure in 59 (52%), acute exacerbation in 31 (27%), and lung cancer in 5 (4%). The median survival from the start of oxygen therapy was 537 ± 74 days. Kaplan–Meier survival curves are displayed in Fig. 1.

### Factors associated with all-cause mortality

Univariable Cox regression analysis demonstrated that lower FVC (*p* < 0.001), lower D_LCO_ (*p* = 0.004), resting hypoxemia (SpO_2_ < 88%) (*p* = 0.006), shorter 6MWT distance (*p* = 0.010), and higher CAT score (*p* < 0.001), higher mMRC scale (*p* = 0.006), and higher GAP stage (*p* < 0.001) were significantly associated with poor prognosis (Table 3). On the other hand, duration of IPF, presence of pulmonary hypertension, and meeting criteria for indication of oxygen therapy were not associated with prognosis. Comorbidities were not associated with prognosis. The study’s criteria, which excluded patients with comorbidities likely to affect survival, may have influenced this result (data not shown). Multivariable analysis revealed that lower BMI (*p* = 0.008), lower FVC (*p* = 0.003), lower D_LCO_ (*p* = 0.030), resting hypoxemia (SpO_2_ < 88%) (*p* = 0.034), lower minimum SpO_2_ on 6MWT (p = 0.004) and higher CAT (*p* = 0.021) were significantly associated with poor prognosis (Table 3). In the other multivariable model, excluding D_LCO_, lower BMI (*p* = 0.028), lower FVC (*p* < 0.001), shorter 6MWT distance (*p* = 0.002), lower minimum SpO_2_ on 6MWT (*p* < 0.001), and higher CAT (*p* = 0.009) were significantly associated with poor prognosis (Table 3).

**Table 3:** Cox proportional hazards regression analyses for prognostic factor.

Factor	Univariable analysis			Multivariable analysis					
				Model 1			Model 2 (without D_LCO_)		
	HR	95%CI	*p*-value	HR	95%CI	*p*-value	HR	95%CI	*p*-value
Age, year	0.998	0.976–1.021	0.856						
Sex, male	1.020	0.633–1.645	0.934						
Body mass index, kg/m^2^	0.968	0.933–1.006	0.095	0.933	0.887–0.982	0.008	0.946	0.901–0.994	0.028
Smoking history	0.697	0.447–1.087	0.112						
Duration of IPF, month	1.001	0.996–1.006	0.844						
Past history of acute exacerbation	1.553	0.988–2.443	0.057	1.533	0.836–2.809	0.167	1.371	0.828–2.270	0.220
FVC, % predicted	0.968	0.955–0.981	< 0.001	0.975	0.959–0.992	0.003	0.973	0.958–0.987	< 0.001
D_LCO_, %predicted	0.980	0.967–0.994	0.004	0.983	0.968–0.998	0.030			
Presence of pulmonary hypertension	1.319	0.897–1937	0.159						
Resting hypoxaemia (SpO_2_ < 88%)	2.015	1.226–3.311	0.006	2.068	1.056–4.052	0.034	1.406	0.811–2.436	0.225
PaCO_2_ breathing air, mmHg	1.007	0.978–1.036	0.642						
6MWT distance, m	0.998	0.997–1.000	0.010	0.999	0.997–1.001	0.286	0.997	0.996–0.999	0.002
Minimum SpO_2_ on 6MWT, %	0.983	0.966–1.002	0.076	0.962	0.937–0.987	0.004	0.955	0.933–0.977	< 0.001
Meet either of the adaptation criteria	1.354	0.928–1.978	0.116						
CAT	1.046	1.020–1.073	< 0.001	1.039	1.006–1.073	0.021	1.039	1.009–1.069	0.009
mMRC scale	1.309	1.080–1.587	0.006						
GAP stage	1.403	1.219–1.616	< 0.001						

HR, hazard ratio; CI, confidence interval; IPF, idiopathic pulmonary fibrosis; FVC, forced vital capacity; D_LCO_, diffusion capacity of the lung for carbon monoxide; SpO_2_, oxygen saturation of peripheral blood; PaCO_2_, arterial partial pressure of carbon dioxide; 6MWT, six-minute walk test; CAT, COPD assessment test; mMRC scale, modified Medical Research Council Dyspnea scale, GAP, gender age and physiology.

## Discussion

This is the first prospective study to investigate the overall survival of patients with IPF after the start of oxygen therapy. In our cohort, the median survival time from the start of oxygen therapy was 17.7 months. Several past retrospective studies reported survival data for patients with interstitial lung disease who started oxygen therapy. Chailleux et al. reported that the prognosis of IPF patients after the start of oxygen therapy was 15–18 months^19^. In a Swedish nationwide study, the median survival time of patients with interstitial lung disease, not limited to IPF, was 8.4 months after the start of oxygen therapy^20^. In a Finnish single-center study, the median survival time of the patients with various interstitial lung diseases was 10.8 months^21^. Previous reports have shown that patients with interstitial lung disease did not survive long after oxygen therapy was initiated, which was also demonstrated in our cohort. In contrast to previous reports, our study is prospective and included only patients with IPF diagnosed according to the 2011 IPF international guidelines. In addition, we excluded patients whose condition was unstable, or who had a comorbidity likely to affect survival at the start of supplemental oxygenation therapy, such as severe heart failure or advanced malignancy.

Although many studies on prognostic factors in IPF have been published, there is a lack of prospective studies conducted from the time that oxygen therapy is started. In this prospective study, low BMI, low FVC, low D_LCO_, low minimum SpO_2_ on 6MWT, and high CAT score were independently associated factors for poor survival. Some studies have suggested that lower BMI may be associated with poor prognosis in IPF^22–25^. This study proved that low BMI is an independent factor for a poor prognosis even in advanced stage IPF. Although FVC is widely recognized as a prognostic factor for IPF, it has not been reported as an independent poor prognostic factor in advanced cases such as those with pulmonary hypertension and those on the waiting list for lung transplantation^26–28^. A possible reason for the discrepancy may be that the FVC percent predicted was about 60% in this study, while it was about 50% in the previously reported advanced cases, indicating that the lung function in our study was still preserved.

In stable IPF, the minimum SpO_2_ on 6MWT is known to be an independent poor prognostic factor^29,30^. In this study, the minimum SpO_2_ on 6MWT was found to be a factor of poor prognosis independent of other poor prognostic factors of poor prognosis including resting hypoxemia. Therefore, the 6-min walk test may be a useful tool to predict prognosis when introducing oxygen therapy to IPF patients.

In this study, we used CAT instead of St. George’s Respiratory Questionnaire (SGRQ) to assess the health status of patients with ILD. The CAT was not developed for patients with IPF, but its scores are strongly correlated with those of the SGRQ, and is a short questionnaire that shows good and valid measurement properties for assessing the health status of patients with ILD^17^. Several previous studies reported that the SGRQ total score was an independent prognostic factor in patients with mild to moderate IPF^31,32^. Although we targeted advanced cases, prognostic factors in this cohort were similar to those reported in non-severe cases.

In recent years, there has been growing recognition that advanced care planning and palliative care are important as an integral part of treating patients with IPF^21,33^. Based on our results, the median survival period after the introduction of oxygen therapy is only about one and a half years, regardless of whether the patient meets the criteria for the introduction of oxygen therapy. Therefore, the initiation of oxygen therapy should be considered a trigger for advance care planning discussions and palliative care consultation, especially in patients with low BMI, low FVC, low D_LCO_, low minimum SpO_2_ on 6MWT, or high CAT score.

There are several limitations to our study. First, we did not validate our results in an external cohort, and so we think further studies are needed to confirm these conclusions. Second, in our cohort, all patients were recruited from Japan only. This data may not be applicable to all countries, as different countries have different criteria for introducing oxygen therapy. Third, we were unable to collect information about medication during the follow-up period. Antifibrotic drugs have been shown to reduce the rate of decline in the FVC and improve prognosis of IPF. In the future, it will be necessary to analyze the effects of antifibrotic drug use in patients with advanced IPF.

In conclusion, the median survival of IPF patients after starting oxygen therapy is only about 1.5 years. Multivariable analysis at the time oxygen therapy is started revealed that low BMI, low FVC, low D_LCO_, low minimum SpO_2_ on 6MWT, and high CAT score were independent factors for a poor prognosis. In addition to pulmonary function tests, 6MWT and patient reported outcomes can be used to predict prognosis more accurately.

### Supplementary Information

Supplementary Table S1.

## Data Availability

The data that support the findings of this study available from the corresponding author upon reasonable request.

## Abbreviations

ACP: Advance care planning
AOT: Ambulatory oxygen therapy
BMI: Body mass index
CAT: COPD assessment test
D_LCO_: Diffusion capacity of the lung for carbon monoxide
FVC: Forced vital capacity
GAP: Gender, age and physiology
IPF: Idiopathic pulmonary fibrosis
IQR: Interquartile range
LTOT: Long-term oxygen therapy
mMRC: Modified Medical Research Council Dyspnea
N/A: Not applicable
P_a_CO_2_: Arterial partial pressure of carbon dioxide
P_a_O_2_: Arterial partial pressure of oxygen
SGRQ: St. George’s respiratory questionnaire
SpO_2_: Oxygen saturation of peripheral blood
TRV: Tricuspid regurgitation peak velocity
6MWT: Six-minute walk test
