# Supplementary material for: Cohort study to evaluate prognostic factors in idiopathic pulmonary fibrosis patients introduced to oxygen therapy
Source: Sci Rep. 2023 Aug 22;13:13664. doi: 10.1038/s41598-023-40508-8 (PMC10444790; doi:10.1038/s41598-023-40508-8)
Supplement: Supplementary file 1 — Supplementary Table S1. [file 41598_2023_40508_MOESM1_ESM.pdf]

Supplementary Table 1.

Comparison of ambulatory oxygen therapy and Long-term oxygen therapy

	Ambulatory oxygen therapy	Long-term oxygen therapy	*p*-value
Patients	61 (41.5)	86 (58.5)	
Patient characteristics at initiation of oxygenation therapy			
Age, year	71.5 (66.0-77.0)	72.0 (66.0-77.0)	NS
Sex, male	47 (77.0)	70 (81.4)	NS
FVC, % predicted	61.7 (54.2-72.4)	61.2 (51.3-72.2)	NS
D_{LCO}, %predicted	40.4 (32.0-53.0)	39.6 (32.0-51.7)	NS
Resting hypoxaemia (SpO_2<88%)	17 (27.9)	5 (5.8)	0.003
P_aCO_2 breathing air, mmHg	40.8 (37.7-45.0)	40.7 (37.6-45.2)	NS
6MWT distance, m	300 (200-425)	282 (190-401)	NS
Lowest SpO_2 on 6MWT, %	82.0 (73.5-87.0)	82.0 (72.0-87.0)	NS
CAT	21.0 (16.0-27.3)	21.0 (15.0-27.0)	NS
mMRC scale			NS
0	2 (2.3)	1 (1.6)	
1	15 (17.4)	3 (4.9)	
2	30 (34.9)	23 (37.7)	
3	30 (34.9)	26 (42.6)	
4	9 (10.5)	8 (13.1)	
GAP stage			NS
I	15 (17.4)	8 (13.1)	
II	70 (81.4)	49 (80.3)	
III	1 (1.2)	4 (6.6)	
Outcome			
Median survival time, days	510 (297-1207)	479 (264-985)	0.041
Death	47	64	NS
Chronic respiratory failure	22 (46.8)	36 (56.3)	
Acute exacerbation	14 (29.8)	16 (25.0)	
Lung cancer	1 (2.1)	4 (6.3)	
Others	10 (21.3)	8 (12.5)	
Lung transplantation	1	0	

Data are presented as median (IQR) or n (%).

Abbreviations: IQR: interquartile range; FVC: forced vital capacity; D_{LCO}: diffusion capacity of the lung for carbon monoxide; GAP: Gender-Age-Physiology; SpO_2: oxygen saturation of peripheral blood; P_aCO_2: arterial partial pressure of carbon dioxide; 6MWT: six-minute walk test; CAT: COPD Assessment Test; mMRC: modified Medical Research Dyspnea.
